# 3D SPECT/CT fusion using image data projection of bone SPECT onto 3D volume-rendered CT images: feasibility and clinical impact in the diagnosis of bone metastasis

**DOI:** 10.1007/s12149-017-1158-3

**Published:** 2017-02-27

**Authors:** Yuji Ogata, Tadaki Nakahara, Kenichi Ode, Yohji Matsusaka, Mari Katagiri, Yu Iwabuchi, Kazunari Itoh, Akira Ichimura, Masahiro Jinzaki

**Affiliations:** Department of Diagnostic Radiology, School of Medicine, Keio University, 35 Shinanomachi, Shinjuku-ku, Tokyo, 160-8582 Japan

**Keywords:** SPECT/CT, Volume rendering, Three-dimensional, Image fusion, Bone metastasis

## Abstract

**Purpose:**

We developed a method of image data projection of bone SPECT into 3D volume-rendered CT images for 3D SPECT/CT fusion. The aims of our study were to evaluate its feasibility and clinical usefulness.

**Methods:**

Whole-body bone scintigraphy (WB) and SPECT/CT scans were performed in 318 cancer patients using a dedicated SPECT/CT systems. Volume data of bone SPECT and CT were fused to obtain 2D SPECT/CT images. To generate our 3D SPECT/CT images, colored voxel data of bone SPECT were projected onto the corresponding location of the volume-rendered CT data after a semi-automatic bone extraction. Then, the resultant 3D images were blended with conventional volume-rendered CT images, allowing to grasp the three-dimensional relationship between bone metabolism and anatomy. WB and SPECT (WB + SPECT), 2D SPECT/CT fusion, and 3D SPECT/CT fusion were evaluated by two independent reviewers in the diagnosis of bone metastasis. The inter-observer variability and diagnostic accuracy in these three image sets were investigated using a four-point diagnostic scale.

**Results:**

Increased bone metabolism was found in 744 metastatic sites and 1002 benign changes. On a per-lesion basis, inter-observer agreements in the diagnosis of bone metastasis were 0.72 for WB + SPECT, 0.90 for 2D SPECT/CT, and 0.89 for 3D SPECT/CT. Receiver operating characteristic analyses for the diagnostic accuracy of bone metastasis showed that WB + SPECT, 2D SPECT/CT, and 3D SPECT/CT had an area under the curve of 0.800, 0.983, and 0.983 for reader 1, 0.865, 0.992, and 0.993 for reader 2, respectively (WB + SPECT vs. 2D or 3D SPECT/CT, *p* < 0.001; 2D vs. 3D SPECT/CT, n.s.). The durations of interpretation of WB + SPECT, 2D SPECT/CT, and 3D SPECT/CT images were 241 ± 75, 225 ± 73, and 182 ± 71 s for reader 1 and 207 ± 72, 190 ± 73, and 179 ± 73 s for reader 2, respectively. As a result, it took shorter time to read 3D SPECT/CT images than 2D SPECT/CT (*p* < 0.0001) or WB + SPECT images (*p* < 0.0001).

**Conclusions:**

3D SPECT/CT fusion offers comparable diagnostic accuracy to 2D SPECT/CT fusion. The visual effect of 3D SPECT/CT fusion facilitates reduction of reading time compared to 2D SPECT/CT fusion.

## Introduction

Metabolic and anatomical information in human organs is often useful to detect potential diseases, evaluate disease conditions, and decide treatment strategies. Nuclear imaging and anatomical imaging modalities have been independently developed until a hybrid imaging system of single-photon emission computed tomography (SPECT), and CT was introduced in 1997 [1]. SPECT/CT system facilitates sequential human data acquisitions of SPECT and CT in an identical position, and the use of SPECT/CT is, therefore, considered to improve the accuracy of image fusion between them. Indeed, image fusion using SPECT/CT is practically useful for detecting bone diseases [2]. Two-dimensional (2D) image interpretation of SPECT/CT fused images (i.e., axial, coronal, and sagittal planes) has generally been performed.

From the standpoint of CT imaging, the bone is a distinct organ in the human body because of its high density, resulting in a marked contrast between the bone and surrounding soft tissue on CT images. Since spiral CT technology allows the generation of three-dimensional (3D) volume-rendered images based on CT voxel value, 3D bone image generation can be attained with simple window level settings. By contrast, bone SPECT as well as planar whole-body bone scintigraphy offers metabolic conditions (i.e., metabolically active or not) in the bone. Thus, an application of volume rendering technique to SPECT/CT image fusion can avoid visualization of extraosseous information, which is similar to the cardiac 3D SPECT/CT fusion in which the left ventricle and coronary vessels are extracted from chest CT data [3, 4]. This technique would make it easier to focus on the evaluation of metabolism and structures in the target organ (e.g., heart or bone) at the same time.

Moreover, one crucial shortcoming of SPECT imaging is that SPECT images have large pixel sizes (4–10 mm) and poor spatial resolution. Therefore, volume-rendered SPECT images are blurred or swollen-shaped, resulting in substantial spillover of bone uptake to surrounding non-osseous tissues on SPECT/CT fused images. To overcome this issue, we developed 3D data expression of bone SPECT into 3D volume-rendered CT images by adapting a commercially available 3D cardiac SPECT/CT fusion tool [3, 4]. 3D-fused images of SPECT and CT with our method are different from the volume-rendered SPECT/volume-rendered CT fused images. The aims of our study were to describe our method and to demonstrate its clinical usefulness.

## Materials and methods

### 3D data expression of bone SPECT into 3D volume-rendered images of CT data (Fig. 1)

**Fig. 1:**
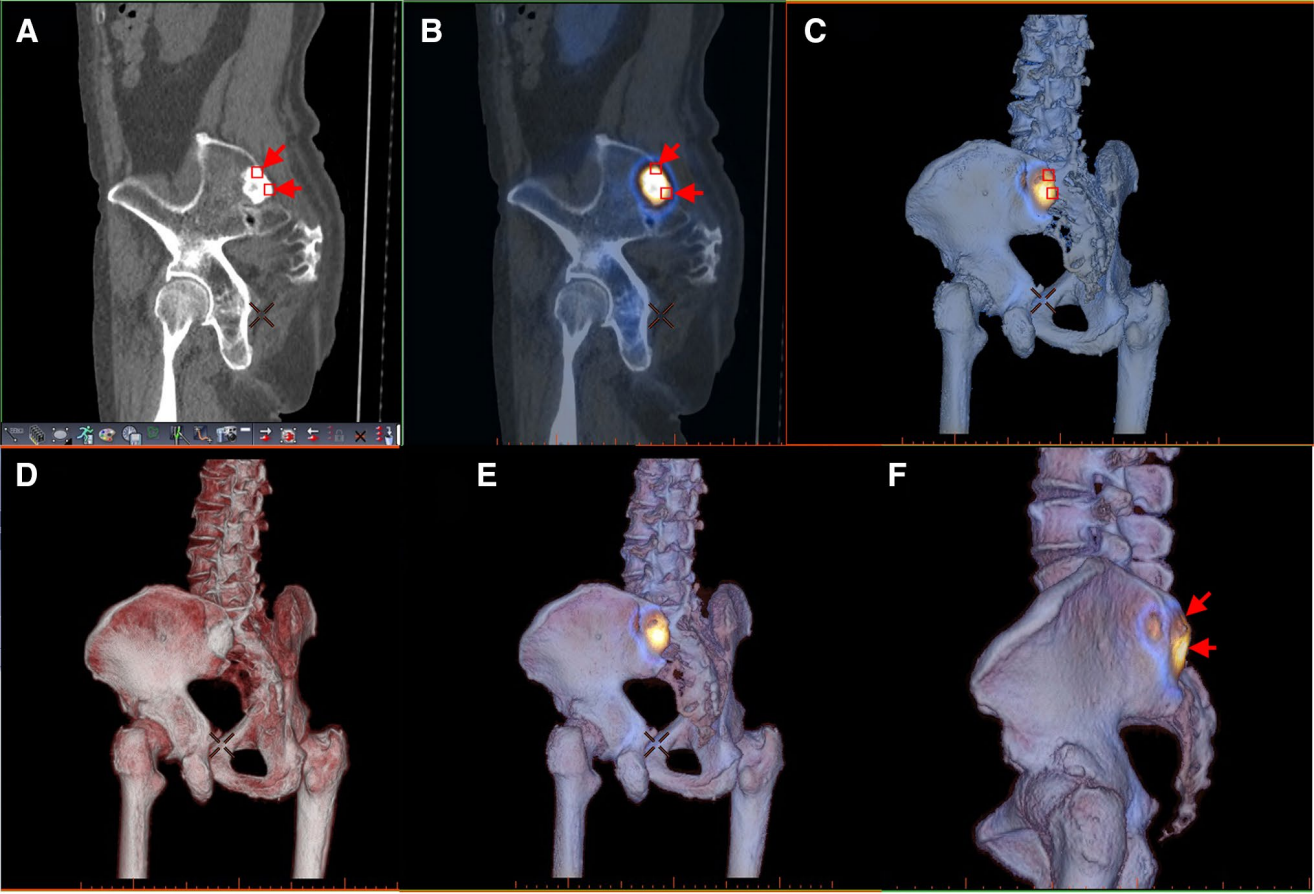
Data expression of bone SPECT into 3D volume-rendered CT images in the patient, as shown in Fig. 1. In the conventional CT, the bone metastasis shows a high density area according to CT values (**a**, *arrows*). In two-dimensional SPECT/CT fusion image, the lesion is colored according to SPECT values at the corresponding site (**b**, *arrows*). In our data expression of bone SPECT into 3D volume-rendered CT images (**c**), three-dimensional bone structures are colored according to SPECT values at the surface of the visualized bones (**a**–**c**, *small squares*). To evaluate CT-based bone structure and bone metabolism, volume-rendered CT image (**d**) is blended with Image C (**e**). No extraosseous uptake is seen with our method (**f**, *arrows*)

Our displaying method was adapted from the cardiac 3D SPECT/CT fusion that was closely described by Gaemperli et al. [4]. In our institution, dedicated cardiac SPECT/CT fusion software (CardIQ Fusion SPECT, GE Healthcare) installed on an image browser (AW server 2, GE Healthcare) is used for this purpose. Unlike the heart, bone structures are rigid and their misalignment between SPECT and CT is expected to be a minimum if patients are instructed to keep stationary. Prior to image fusion, the semi-automatic extraction of bone structures is performed on the basis of CT values on our image workstation. During this process, the urinary tracts and bladder are automatically eliminated. To create the 3D bone metabolic images, the 3D cardiac SPECT/CT fusion tool was used; SPECT data are simply expressed in color at the corresponding location on 3D volume-rendered CT images.

Therefore, the two types of 3D colored images can be generated: bone structural images colored according to CT voxel values (conventional volume rendering) and bone metabolic images colored according to SPECT voxel values. Then, these two images are blended, so that the resultant 3D images facilitate simultaneous evaluation of bone metabolism and anatomy. Since apparent voxel size of the blended images is equivalent to that of volume-rendered CT, they provide a visual effect similar to that of contrast enhancement of the bone on CT or magnetic resonance imaging.

**Fig. 2:**
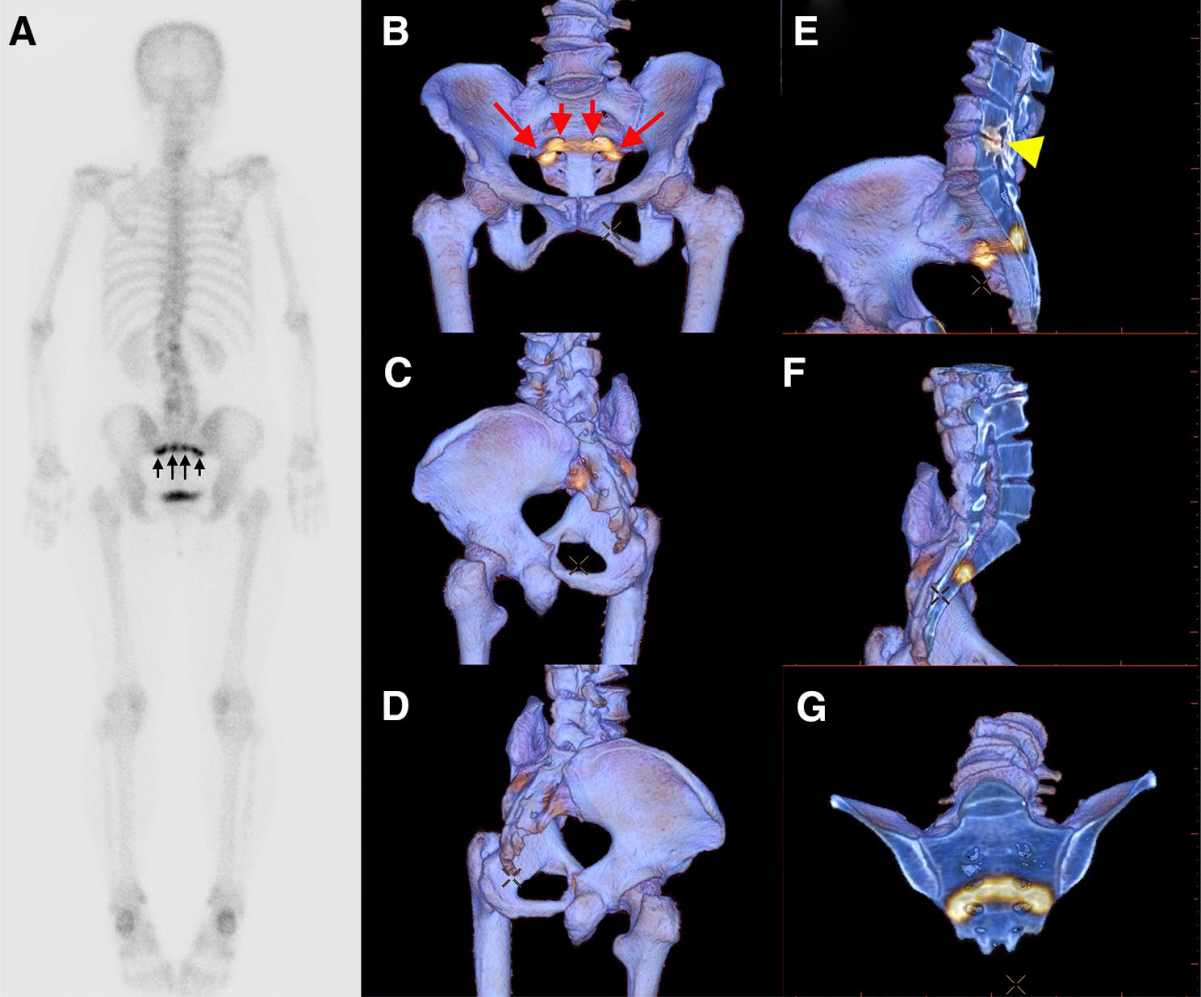
Application of “clip-plane editing” to our data expression of bone SPECT into 3D volume-rendered CT images. Whole-body two-dimensional scintigraphy in a breast cancer patient shows band-like uptake in the sacrum (**a**, *arrows*). The three-dimensional fused images with our method show that the uptake is mainly observed in the anterior side of the sacrum (**b**–**d**, *arrows*). Cross-sectional images of the 3D data show that the uptake is not tumor-like, making the diagnosis of insufficiency sacral fracture (**e**–**g**). In addition, osteoarthrosis around the joint between the fourth and fifth lumbar spine with increased metabolic activity is seen (**e**, *arrowhead*)

Opacity for bone structures on the 3D volume-rendered images is basically set at 100%, so that metabolic data are visualized only on the surface of the displayed bone (Fig. 1). When evaluating internal metabolisms and structures of the bones, we employ a technique to cut out the volume data, which is called a clip-plane editing [5], to further observe their cross-sectional images (Fig. 2).

### Clinical applications of 3D SPECT/CT fusion to bone imaging

From May 2013 to July 2014, whole-body bone scintigraphy with technetium-99m hydroxymethylene diphosphonate (Tc-99m HMDP) showed hypermetabolic sites in 318 patients. These patients underwent this imaging test because of initial staging (*n* = 82) and suspected bone metastasis (*n* = 236). To further evaluate the abnormal sites, bone SPECT and CT using integrated SPECT/CT systems (Discovery NM/CT 670pro, GE Healthcare) consisting of SPECT scanner and 16-slice multidetector CT were performed in the areas (1–3 SPECT steps) covering the hypermetabolic sites. SPECT/CT scans were performed as a part of bone scintigraphic test or dual tests of bone scintigraphy and diagnostic CT. Regarding the bone scintigraphic test alone, low-dose CT for attenuation correction was added to bone SPECT and used for image fusion. Image noise in low-dose CT is improved with recently developed iterative reconstruction algorithms (about 1 mGy of volume CT dose index (CTDIvol)) while maintaining its image contrast. Regarding the dual tests, diagnostic CT was also performed just after the acquisition of bone SPECT in an identical position (5–13 mGy of CTDIvol).

### Clinical evaluation of our 3D display method

Our 3D display method simply erases from the images which results in some kind of information loss and worsens the image statistics compared to conventional 2D SPECT/CT fusion. Therefore, one of the objectives in the present study were to elucidate the non-inferiority of 3D SPECT/CT over the conventional 2D SPECT/CT in the diagnosis of bone metastasis.

Whole-body scintigraphy and SPECT (WB + SPECT), conventional 2D SPECT/CT fusion, and our 3D SPECT/CT fusion in 318 patients were evaluated by two independent board-certified radiologists with 3-year (reader 1) and 8-year (reader 2) experiences in SPECT/CT fusion imaging. Each of the three image sets was reviewed with an interval of more than 3 months. The method of comparing diagnostic performances between these images was similar to that described by elsewhere [6, 7]. Briefly, each of the lesions with abnormal tracer uptake in SPECT was classified using a four-point diagnostic confidence scale:

1. Definitely benign;
2. Possibly benign;
3. Possibly malignant;
4. Definitely malignant.

Scoring criteria were based on the location and intensity of tracer uptake [8–10] and CT appearance [2, 6]; joint uptake, tandem rib uptake, and linear vertebral or sacral uptake were considered to be degenerative joint disease, traumatic change and compression or insufficiency fracture, respectively. Increased tracer uptake with random distribution, intense pedicle uptake, and dough-nut shaped uptake was all considered metastatic. On CT, the presence of bone cyst, bone island, Schmorl’s node, osteophyte, arthropathy-related changes, enthesopathy, traumatic changes, and compression fracture was regarded benign. By contrast, the presence of lytic, sclerotic, or mixed lytic-sclerotic changes that could not be explained by these benign changes was considered malignant.

The diagnosis of bone metastasis or benign nature was confirmed by subsequent bone scans with a follow-up of more than 1 year, spine MRI and FDG PET. Bone metastasis by bone biopsy was confirmed only in one case. A benign lesion with the score of 1 or 2 was considered true negative. A metastatic lesion with the score of 3 or 4 was considered true positive [7].

The time needed for scoring the lesions in each patient was recorded for WB + SPECT, 2D SPECT/CT, and 3D SPECT/CT, respectively. In other words, the duration of interpreting images was measured; it did not include the duration of image processing. Regarding the interpretation of 3D SPECT/CT images, it should be noted that an area of increased Tc-99m HMDP uptake is frequently seen in the medial side of the pubis without any causes of increased bone metabolism. This is due to photon scattering from high urinary 99mTc-HMDP accumulation. This finding was considered to be an artifact in this study.

The institutional review board of our hospital granted permission for this retrospective review of the imaging and clinical data, and waived the need for obtaining informed consent from the patients.

### Statistical analysis

The weighted kappa statistic was used to calculate inter-observer agreement in the diagnosis of bone metastasis in which kappa was classified as follows [11]: 0, poor agreement; <0.20, slight agreement; 0.21–0.40, fair agreement; 0.41–0.60, moderate agreement; 0.61–0.80, substantial agreement; and 0.81–1.00, almost perfect agreement.

Receiver operating characteristic (ROC) curve analysis was performed to compare the diagnostic performance between WB + SPECT, 2D SPECT/CT, and 3D SPECT/CT in assessing bone metastasis.

Paired Student *t* tests for time difference in interpreting images on a per-patient basis were performed between WB + SPECT, 2D SPECT/CT, and 3D SPECT/CT. A Bonferroni adjusted *p* value <0.05 was considered significant.

## Results

Patient characteristics are shown in Table 1. In 308 cases, bone metastasis or benign nature was confirmed by follow-up bone scan or as a result of spine MRI or FDG PET. Bone biopsy was performed in the remaining ten cases. One, two and three steps SPECT were performed in 206, 92, and 10 cases, respectively. Low-dose CT for attenuation correction of SPECT and diagnostic CT were performed in 281 and 37, respectively. In this population, 1746 abnormal metabolic sites consisting of 744 bone metastases and 1002 benign lesions were evaluable for diagnostic comparison (Table 2).

**Table 1:** Patient characteristics (*n* = 318)

	*n* (%)
Demographics	
Age(years)	65 ± 13
Gender	
Male	103 (32)
Female	215 (68)
Disease	
Breast cancer	130 (41)
Lung cancer	92 (29)
Prostate cancer	39 (12)
Renal cancer	23 (7)
Esophageal cancer	14 (4)
Hepatic cancer	6 (2)
Gastric cancer	5 (2)
Colon cancer	3 (1)
Bladder cancer	3 (1)
Head and neck cancers	3 (1)
CT protocol	
For attenuation correction (low-dose)	281 (88)
For diagnosis	37 (12)
Bone metastasis	
Present	161 (51)
Absent	157 (49)

Values are n, mean ± SD, or *n* (%)

**Table 2:** Evaluable abnormal uptake sites (*n* = 1746)

	*n* (%)
Bone metastasis (*n* = 744)	
Spine	298 (17)
Pelvis	171 (10)
Rib	153 (9)
Others	122 (7)
Benign etiology (*n* = 1002)	
Degenerative joint disease	892 (51)
Traumatic change/fracture	105 (6)
Others	5 (0)

Values are *n*, or *n* (%)

### Practicability of 3D SPECT/CT

Data expression of bone SPECT into 3D volume-rendered CT images was successful for all patients. In most cases, the process of our 3D image generation took about 5 min on average by experienced operators. However, additional manual procedure was required for properly extracting bone tissues from low-dose CT data due to decreased bone density in 26 cases with severe osteoporosis (8.2%) and 17 obese patients (5.3%); since the extraction process is dependent on tissue contrast in CT images, it was quite difficult to selecting the entire bone structures at one step. Therefore, the threshold for extracting bone surfaces was manually determined according to the CT value of the bone cortex in an individual case. Then, “Close Holes” technique was applied to re-fill the extracted data that are encompassed with the bone surfaces [12].

### Diagnostic analysis of WB + SPECT, 2D SPECT/CT, and 3D SPECT/CT

The results of image interpretation for the two independent readers were shown in Table 3. Among 1746 lesions, there were 1250 equivocal judgments (72%) (i.e., ‘possibly benign’ or ‘possibly malignant’) for reader 1 and 1228 (70%) for reader 2 when interpreting WB + SPECT images. By contrast, there were 336 (19%) and 340 (19%) for reader 1 and 284 (16%) and 278 (16%) for reader 2 when interpreting 2D SPECT/CT and 3D SPECT/CT images, respectively. As a result, sensitivity, specificity, and positive and negative predictive values were higher for 2D or 3D SPECT/CT than those for WB + SPECT (Table 4). Although there was substantial agreement (k = 0.72) between the two readers even in WB + SPECT, 2D (*k* = 0.90), or 3D SPECT/CT (*k* = 0.89) reached better agreement between them.

**Table 3:** Diagnostic confidence of WB and SPECT, 2D SPECT/CT, and 3D SPECT/CT for two independent readers

	WB and SPECT		2D SPECT–CT		3D SPECT–CT	
	Metastasis (*n* = 744)	Benign (*n* = 1002)	Metastasis (*n* = 744)	Benign (*n* = 1002)	Metastasis (*n* = 744)	Benign (*n* = 1002)
Reader 1						
Definitely benign	23	245	15	914	13	906
Possibly benign	140	527	52	74	52	78
Possibly malignant	401	182	198	12	194	16
Definitely malignant	180	48	479	2	485	2
Reader 2						
Definitely benign	8	271	4	939	3	942
Possibly benign	126	570	33	54	34	49
Possibly malignant	390	142	191	6	187	8
Definitely malignant	220	19	516	3	520	3

**Table 4:** Diagnostic accuracy of WB and SPECT, 2D SPECT/CT, and 3D SPECT/CT for two independent readers and inter-reader variability

	Reader 1				Reader 2				Inter-reader variability*
	Sens (%)	Spec (%)	PPV (%)	NPV (%)	Sens (%)	Spec (%)	PPV (%)	NPV (%)	
Renal, esophageal and hepatic cancers (*n* = 43)									
WB and SPECT	20	74	29	64	34	85	55	71	0.66
2D SPECT–CT	78	98	95	89	90	99	97	95	0.88
3D SPECT–CT	79	99	97	90	90	99	97	95	0.89
The other cancers (*n* = 275)									
WB and SPECT	85	78	75	87	87	84	81	91	0.82
2D SPECT–CT	93	99	98	94	96	99	99	97	0.91
3D SPECT–CT	93	98	97	95	96	99	99	97	0.91
Total (*n* = 318)									
WB and SPECT	78	77	72	83	82	84	79	86	0.72
2D SPECT–CT	91	99	98	94	95	99	99	96	0.90
3D SPECT–CT	91	98	97	94	95	99	98	96	0.89

*Sens* sensitivity, *Spec* specificity, *PPV* positive predictive value, *NPV* negative predictive value

^*^ Weighted kappa value based on the four-point scale

Regarding the differences in diagnostic accuracy among malignant tumors, WB + SPECT showed much lower sensitivity in renal, esophageal, and hepatic cancers than in the other malignancies for both readers (*p* < 0.001) (Table 4). These cancers mostly had osteolytic metastases in the present study. Although 2D SPECT/CT significantly improved sensitivity in these cancers, the sensitivity was still low compared to the other malignancies for both readers (*p* < 0.01). In addition, 3D SPECT/CT showed similar tendency for both readers (*p* < 0.01). There was no significant difference in specificity among malignant tumors.

**Fig. 3:**
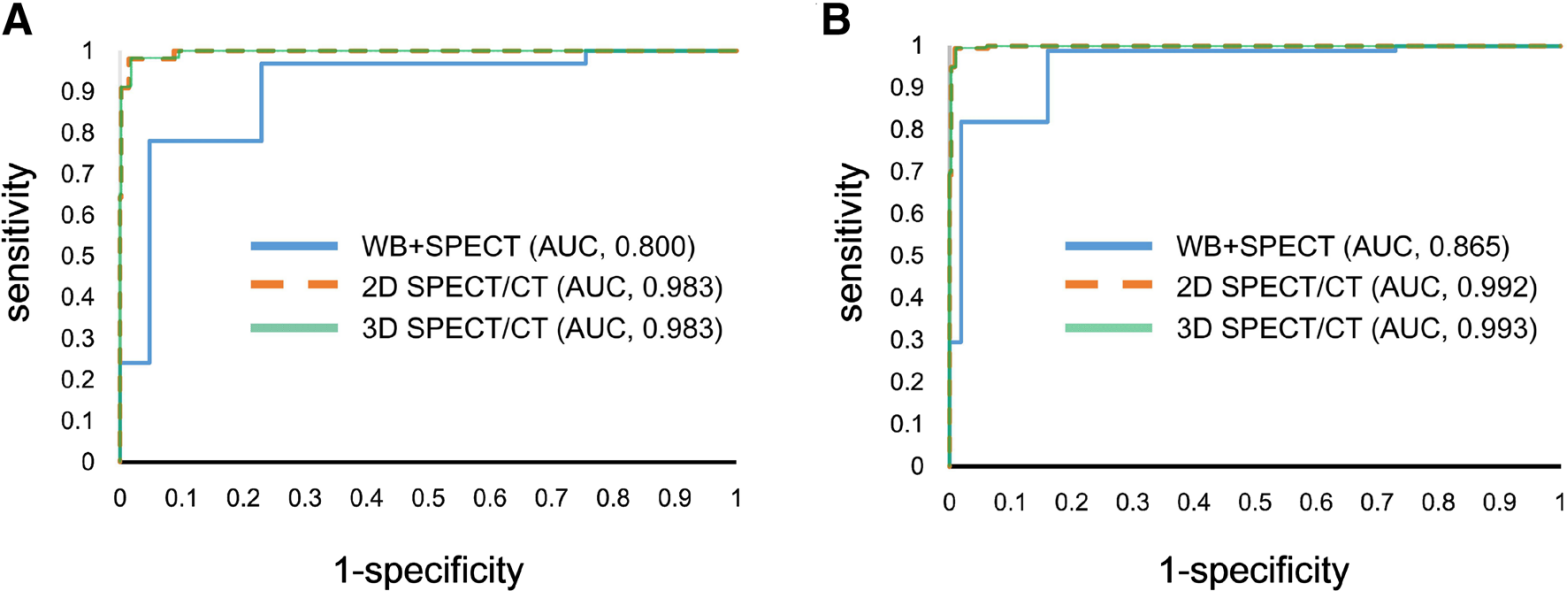
ROC curves with AUC values for the diagnosis of bone metastasis on WB + SPECT, 2D SPECT/CT fusion, and 3D SPECT/CT fusion images by reader 1 (**a**) and 2 (**b**). For both readers, the AUC value on 2D SPECT/CT fusion or 3D SPECT/CT fusion was significantly higher than that on WB + SPECT (*p* < 0.001), whereas there was no significant difference between 2D SPECT/CT fusion and 3D SPECT/CT fusion (*p* = 0.6239 for reader 1; *p* = 0.5076 for reader 2)

The changes in sensitivity and specificity in relation to the applied cut-off scores are shown in Fig. 3. ROC analyses showed that WB + SPECT, 2D SPECT/CT, and 3D SPECT/CT had an area under the curve of 0.800, 0.983, and 0.983 for reader 1, 0.865, 0.992, and 0.993 for reader 2, respectively (WB + SPECT vs. 2D or 3D SPECT/CT, *p* < 0.001; 2D vs. 3D SPECT/CT, n.s.).

### Time for interpreting WB + SPECT, 2D SPECT/CT, and 3D SPECT/CT images

**Fig. 4:**
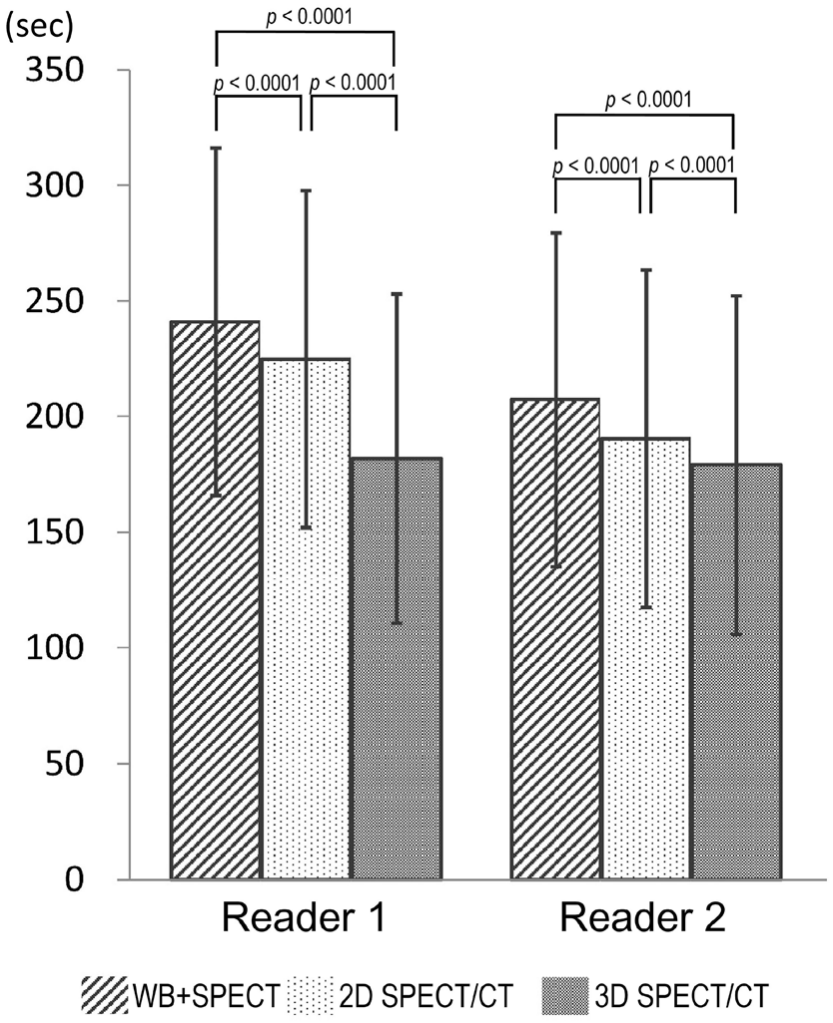
Differences in the duration of reading images between WB + SPECT, 2D SPECT/CT fusion, and 3D SPECT/CT fusion

Time required for scoring the lesions with abnormal tracer uptake in 318 patient was measured for WB + SPECT, 2D SPECT/CT, and 3D SPECT/CT (Fig. 4). The durations of interpretation of WB + SPECT, 2D SPECT/CT, and 3D SPECT/CT images were 241 ± 75, 225 2573, and 182 ± 71 s for reader 1 and 207 ± 72, 190 ± 73, and 179 ± 73 s for reader 2, respectively. As a result, it took shorter time to read 3D SPECT/CT images than 2D SPECT/CT (*p* < 0.0001) or WB + SPECT images (*p* < 0.0001). In particular, the time needed for the interpretation of 3D SPECT/CT images was 25 and 19% shorter than that of WB + SPECT and 2D SPECT/CT images, respectively.

### Case presentations

**Fig. 5:**
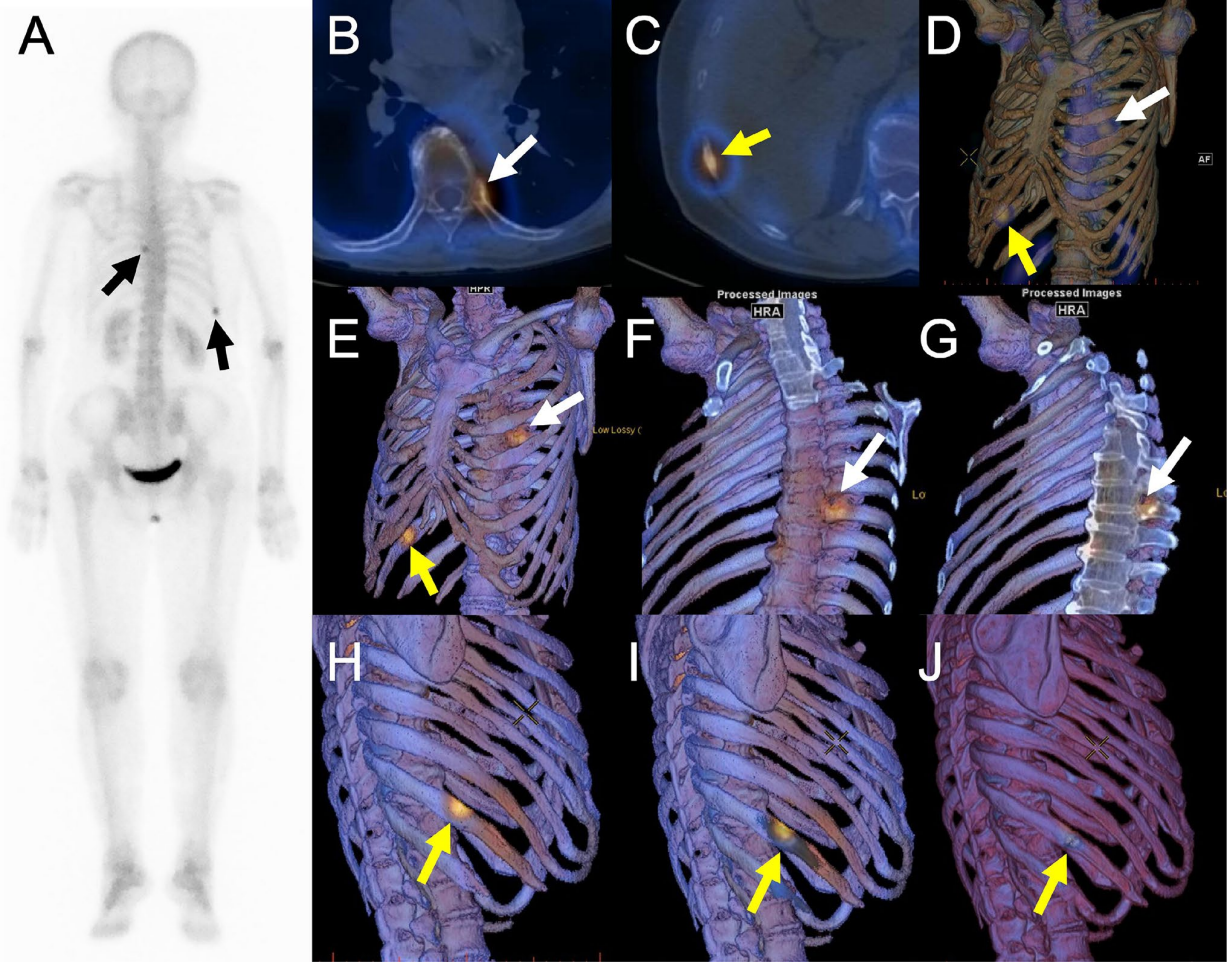
Whole-body scintigraphy (**a**), 2D SPECT/CT fusion (**b** and **c**), 3D SPECT/CT fusion using volume-rendered SPECT images (**d**) or image data projection of bone SPECT onto 3D volume-rendered CT images without (**e, h**, and **j**) or with clip-plane editing (**f, g**, and **i**) in a breast cancer patient with co-existence of bone metastasis and fracture in the ribs. Osteolytic change is clearly seen in 2D SPECT/CT (**b**) and our 3D SPECT/CT (**f** and **g**) images in the proximal site of the left eighth rib (*white arrows*). By contrast, a fracture line is shown at the hypermetabolic site in 3D SPECT/CT fusion (**h**–**j**)

Figure 5 shows WB, 2D SPECT/CT fusion, and 3D SPECT/CT fusion using volume-rendered SPECT images or image data projection of bone SPECT onto 3D volume-rendered CT images (our method) in a breast cancer patient with co-existence of bone metastasis and fracture in the ribs. Whole-body scintigraphy shows two hypermetabolic sites in the ribs (black arrows). Both readers 1 and 2 correctly rated a hypermetabolic site in the proximal site of the left eighth rib (yellow arrows in the SPECT/CT images) as definitely malignant because there were no other reasons for osteolytic change than metastasis. On the other hand, readers 1 and 2 rated a lesion in the right tenth rib as possibly malignant and possibly benign when interpreting 2D SPECT/CT images, respectively. However, this lesion was reported as definitely benign in both readers after 3D SPECT/CT fusion images were reviewed, because a fracture line was recognized in the craniocaudal direction (Fig. 5j). In this case, the follow-up SPECT/CT 1 year after the initial SPECT/CT confirmed the diagnoses of a bone metastasis in the left eighth rib and a benign fracture in the right tenth rib. Although a slight misregistration was found between the hypermetabolic center in SPECT and the fracture in CT, the fracture was considered to be responsible for increased bone metabolism.

**Fig. 6:**
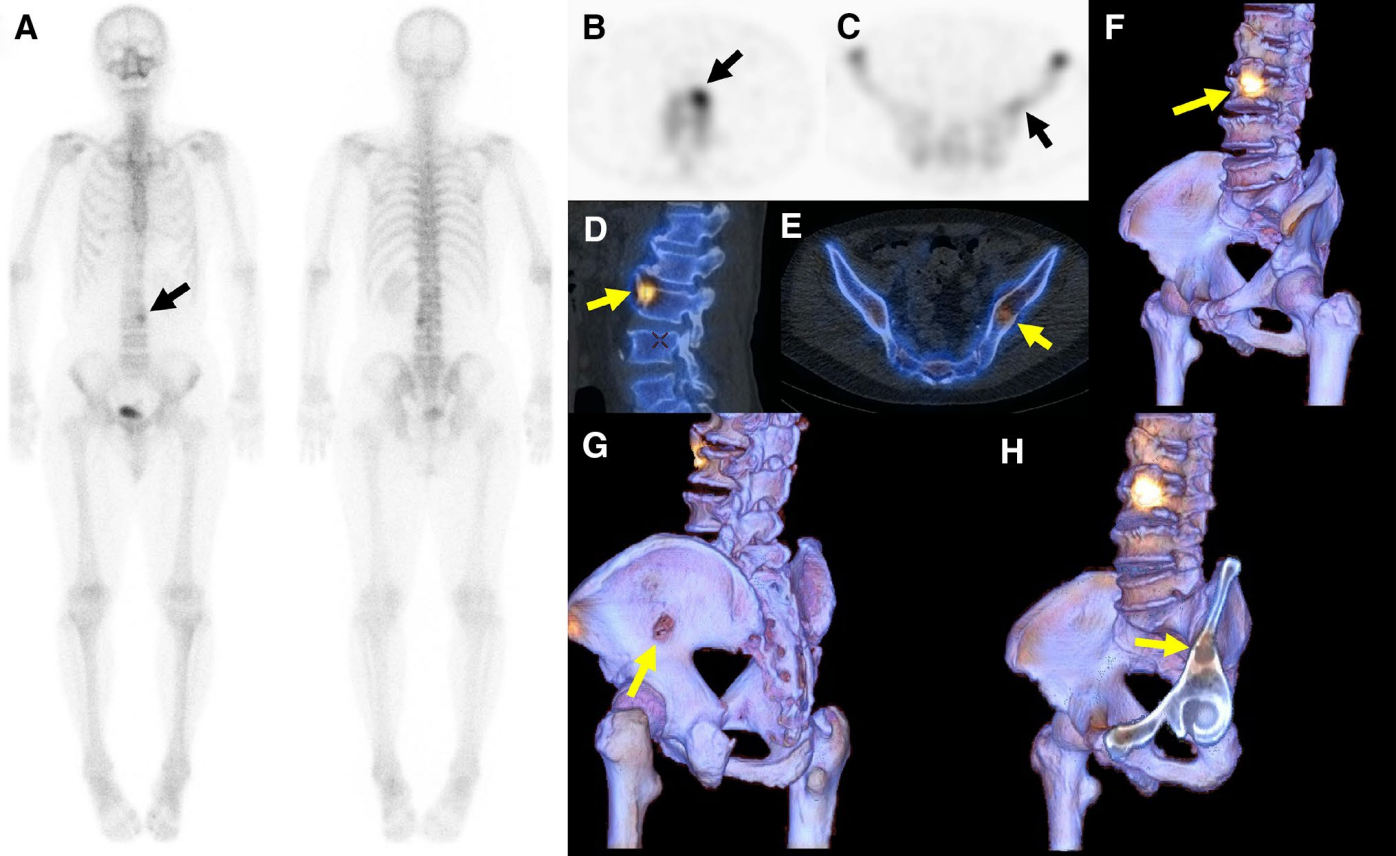
Whole-body scintigraphy (**a**), SPECT (**b** and **c**), 2D SPECT/CT fusion (**d** and **e**), and image data projection of bone SPECT onto 3D volume-rendered CT images without (**f** and **g**) or with clip-plane editing (**h**) in a renal cancer patient with co-existence of an osteolytic metastasis in the left iliac bone and a hypermetabolic osteophyte in the third lumbar vertebra. The iliac lesion was barely visible in whole-body scintigraphy (**a**) and SPECT showed faint uptake (**c**), suggesting benign nature rather than metastasis. Osteolytic change is clearly seen in 2D SPECT/CT (**e**) and our 3D SPECT/CT shows a hole at the slightly hypermetabolic site (**g**). Clip-plane method allows the visualization of an osteolytic lesion with minimal pathological fracture (**h**)

Figure 6 shows WB + SPECT, 2D SPECT/CT fusion, and 3D SPECT/CT fusion in a renal cancer patient with co-existence of an osteolytic metastasis in the iliac bone and a hypermetabolic osteophyte in the lumbar spine. Whole-body scintigraphy shows only one hypermetabolic site (black arrow in Fig. 6a), whereas SPECT additionally shows a slightly hypermetabolic site in the left iliac bone (black arrow in Fig. 6c). Both readers 1 and 2 rated a vertebral lesion as possibly malignant and an iliac lesion as possibly benign when reading WB + SPECT images. However, the vertebral lesion was reported as definitely benign in both readers after reviewing 2D or 3D SPECT/CT fusion images, because a prominent osteophyte was recognized at the hypermetabolic site (Fig. 6d, f). In addition, osteolytic change was clearly seen in the iliac lesion in 2D or 3D SPECT/CT, allowing both readers to make a correct diagnosis of bone metastasis. In the review of 3D SPECT/CT images, it was very easy to detect a hole in the left iliac bone (Fig. 6g). After the clip-plane editing, an osteolytic metastasis with a cortical irregularity (yellow arrow) was revealed (Fig. 6h).

## Discussion

In the present article, our novel technique of 3D SPECT/CT fusion was shown together with our clinical experience. To our knowledge, prototypic 3D volume rendering, which is one of the 3D imaging techniques, was applied to medical imaging more than 40 years ago [13]. Since then, the improvement of volume rendering techniques as well as the development of image processing hardware and software makes volumetric visualization of radiological data more practical and reliable [14]. Indeed, since multislice CT allows to obtain the enormous size of the imaging data sets in a short time, 3D imaging techniques have been utilized as a part of image interpretation or sometimes to omit the time-consuming review of axial images in daily clinical practice. In the field of nuclear medicine, 3D cardiac SPECT/CT fusion is still progressing as we reported the new method of exhibiting the 3D cardiac imaging [15]. The present study on the application of 3D imaging to bone SPECT revealed that 3D SPECT/CT had a high performance comparable to 2D SPECT/CT in the diagnosis of bone metastasis. In addition, the time taken for reading 3D SPECT/CT images was significantly shorter than that for reading 2D SPECT/CT images.

In general, bone SPECT/CT is performed to evaluate bone function and anatomy, although CT as a part of the SPECT/CT examinations exhibits not only bone structures but any other organs. Our technique is developed based on the fact that 3D cardiac SPECT/CT fusion technique eliminates the non-cardiac anatomic data during image generation in order to focus on the target organ. Therefore, we first applied volume rendering to both SPECT and CT, as mentioned in Introduction. However, this method was unacceptable to be used in clinical practice due to poor spatial resolution and large matrix size in SPECT. As a result, we devised an alternative method for expressing bone SPECT data onto 3D volume-rendered CT images. With our method, bone metabolism is colored onto the bone structural images, which gives the visual effect that apparent matrix size for SPECT is the same as that for CT. In addition, spillover of Tc-99m HMDP uptake to the surrounding soft tissue on 2D SPECT/CT images is not visualized with our 3D SPECT/CT method. Thus, our 3D fusion images resemble conventional 3D volume-rendered CT images that referral physicians are familiar with. Although our 3D image generation requires a specific image workstation and takes much time in cases with severe osteoporosis, there were no technical errors in this study.

Many studies have demonstrated the usefulness of SPECT/CT in the diagnosis of bone metastasis [2, 6, 7, 16, 17]. Zhang et al. compared the diagnostic accuracy of SPECT and SPECT/CT using a four-point scale [6], reporting that 67.9% of lesions were equivocal (score 2–3) by SPECT, but only 19.6% were equivocal by SPECT/CT, which is very similar to our results. In a study by Romer et al. [17], SPECT/CT allowed to sort more than 90% of intermediate findings into benign or malignant bone lesions. Very high inter-reader agreement was achieved using a four-point scale for SPECT/CT in some previous studies [6, 7] and ours (*k* = about 0.90). Palmedo et al. reported that SPECT/CT contributed to proper downstaging or upstaging without further diagnostic procedures as a result of increased diagnostic accuracy [16]. Interestingly, reading SPECT/CT fused images offered higher diagnostic performance than separate reading of SPECT and CT [2].

In our study, WB+SPECT showed poor sensitivity (Table 4) compared to the aforementioned conventional studies which mainly enrolled prostate, lung or breast cancer patients. By contrast, renal, esophageal and hepatic cancers have been scarcely included. The difference in patient population would account for lower sensitivity in our study, because osteolytic metastasis, which was mostly seen in renal, esophageal, and hepatic cancers, was difficult to be interpreted as malignant without morphological information. Indeed, there were not only the differences in patient population, but also readers’ experience of SPECT and diagnostic CT, or image analysis between our study and the conventional studies; nevertheless, SPECT/CT had a substantial impact on increasing the diagnostic confidence and accuracy compared to SPECT alone. .

Time taken for reading 3D SPECT/CT images was shorter than that for 2D SPECT/CT images, while 3D SPECT/CT fusion did not prove a diagnostic inferiority over 2D SPECT/CT fusion in the present study. Since structural information outside the bone is probably unnecessary for the diagnosis of bone metastasis, 3D SPECT/CT fusion might be an alternative method to display bone metabolism and structures. An advantage of 3D images is to be able to understand whole anatomical information at first sight, as shown in case presentations. We believe that 3D SPECT/CT is more convincing than 2D fusion for young radiologists with little experience in CT, referral physicians, or even patients. However, since the presented data were scored by the experienced radiologists, but not by the referring clinicians, the difference in clinical impact between 2D and 3D SPECT/CT by non-radiologists is unknown. It was technically difficult to instruct the referring clinicians on how to use our image workstation. Regarding the potential applications of bone 3D SPECT/CT, we have reported its clinical usefulness in evaluation of mandibular osteomyelitis [18]. 3D SPECT/CT might be useful to decide the location for appropriate bone biopsy. The applications of 3D fusion to such specific objectives will require substantial more evaluation.

It seems clinically practical to generate our 3D SPECT/CT fusion images, because it took only about 5 min using the dedicated fusion software and workstation in more than 80% of the cases. However, it was not easy to extract bone tissue at one step in osteoporotic or obese patients. At present, this is a vexing problem when creating 3D volume rendering images. It is also important for operators to be able to perform the automatic and manual editing of CT volume data and understand those effects on 3D image display. In this regard, the improvement of bone segmentation technique and reconstruction algorithm for noise reduction in a CT portion of SPECT/CT will overcome these issues.

Our study has some limitations. First, it was a retrospective study and we did not review any metastatic lesions without detectable tracer uptake in SPECT. Therefore, the diagnostic performances for WB + SPECT, 2D SPECT/CT, and 3D SPECT/CT would be all overestimated in the present study. Second, almost all bone lesions were not histopathologically proven, which is one of the biggest limitations. In our clinical settings, follow-up SPECT/CT was of great value for the confirmation of bone metastasis by careful comparison of CT appearance and bone metabolism in both the previous and follow-up SPECT/CT. In addition, MRI or FDG PET also served as reference standards in some cases in which SPECT/CT showed equivocal results. Finally, although the bone is a rigid organ, a misregistration between SPECT and CT in the rib can occur due to respiratory motion (Fig. 5). However, we believe that these limitations did not deteriorate the value of our results in this comparative assessment.

## Conclusion

We developed a method of 3D SPECT/CT fusion display using image data projection of bone SPECT onto 3D volume-rendered CT images. 3D SPECT/CT fusion provides as high reproducibility and accuracy for diagnosing bone metastasis as 2D SPECT/CT. Although there are still some problems generating 3D SPECT/CT fusion images, 3D SPECT/CT fusion images reduced the interpretation time compared to 2D SPECT/CT fusion images.
